# Precision Mapping of a Maize MAGIC Population Identified a Candidate Gene for the Senescence-Associated Physiological Traits

**DOI:** 10.3389/fgene.2021.716821

**Published:** 2021-10-04

**Authors:** Marlon Caicedo, Eduardo D. Munaiz, Rosa A. Malvar, José C. Jiménez, Bernardo Ordas

**Affiliations:** ^1^Instituto Nacional de Investigaciones Agropecuarias (INIAP), Quito, Ecuador; ^2^National Research Council of Spain (CSIC) Misión Biológica de Galicia, Pontevedra, Spain; ^3^National Institute of Forestry, Agriculture and Livestock Research (INIFAP), Cuauhtémoc, Mexico

**Keywords:** maize, senescence, chlorophyll index, physiological, phenotyping, MAGIC, GWAS

## Abstract

Senescence is an important trait in maize (*Zea mais* L.), a key crop that provides nutrition values and a renewable source of bioenergy worldwide. Genome-wide association studies (GWAS) can be used to identify causative genetic variants that influence the major physiological measures of senescence, which is used by plants as a defense mechanism against abiotic and biotic stresses affecting its performance. We measured four physiological and two agronomic traits that affect senescence. Six hundred seventy-two recombinant inbred lines (RILs) were evaluated in two consecutive years. Thirty-six candidate genes were identified by genome-wide association study (GWAS), and 11 of them were supported by additional evidence for involvement in senescence-related processes including proteolysis, sugar transport, and sink activity. We identified a candidate gene, Zm00001d043586, significantly associated with chlorophyll, and independently studied its transcription expression in an independent panel. Our results showed that Zm00001d043586 affects chlorophyl rate degradation, a key determinant of senescence, at late plant development stages. These results contribute to better understand the genetic relationship of the important trait senescence with physiology related parameters in maize and provide new putative molecular markers that can be used in marker assisted selection for line development.

## Introduction

Genetic studies of delay of leaf senescence indicate that it is a multigenic trait (Guiboileau et al., 2010; Kant et al., 2015), as a result of the interacting of several metabolic paths that involve nutrient absorption and remobilization (Hörtensteiner and Feller, 2002; Lim and Nam, 2005), and the disassembly of the photosynthesis apparatus (Balazadeh, 2014). These biochemical processes of chlorophyll breakdown during leaf senescence lead to physiological changes in the plant phenotype (Balazadeh, 2014). Phenotypic changes due to delay senescence has been described in several species, e.g., *Zea mais L.* (Gentinetta et al., 1986; Zhang et al., 2019), *Oryza sativa* (Ramkumar et al., 2019), or *Glycine max* (Guiamét et al., 1990). Delay of senescence has a favorable influence on crop production: positively associated with plant lodging resistance (Duvick and Cassman, 1999; Munaiz et al., 2021), increasing biomass yield (Borrell et al., 2000), favoring grain yield in plants grown under drought conditions (Borrell et al., 2014), and heat stress tolerance (Pinto et al., 2016). One important aspect is that late senescence allows for a longer photosynthesis period during the life cycle of the plant and larger dry matter accumulation during grain filling, leading to crops with higher yield performance (Valentinuz and Tollenaar, 2004).

Quantitative trait loci (QTL) have been identified using traditional linkage mapping, multiparent advanced generation intercrosses (MAGIC) populations and association mapping in diverse panels. MAGIC’s advantage compared to biparental populations is the possibility of simultaneous studies of more than two alleles, usually eight alleles (Valdar et al., 2006). Traditionally, QTL mapping has used biparental populations, e.g., Glowinski and Flint-Garcia (2018); however, multiple inbred founder methods, which directionally intermate several times to obtain a unique combination of the genetic material from all founders in a single inbred line with a unique mosaic of small allelic haplotypes, have been used recently. Thus, providing sufficient genetic base, the higher number of parents and recombination events of the MAGIC population are advantageous in comparison with biparental population, while keeping pedigrees and genetic structure known in both cases. The first one allows for higher allelic diversity, whereas the later can benefit from having smaller haplotypes regions. The main limitation of biparental QTL methods is the reduce resolution, primarily because of the smaller number of crossing-over events accumulated over a few generations. On the other hand, diverse association panels made up of nonrelated genotypes that have accumulated a higher number of recombination events since the last shared progenitor could increase this resolution and, thus, display a more restricted linkage disequilibrium between pairs of neighboring molecular markers (Hyten et al., 2007) benefiting of a larger number of marker density and coverage of the entire genome.

MAGIC populations have been used in QTL identification analysis in a wide range of crops including rice (Bandillo et al., 2013), wheat (Huang et al., 2012), fava bean (Sallam and Martsch, 2015), maize (Dell’Acqua et al., 2015), barley (Sannemann et al., 2018), and sorghum (Ongom and Ejeta, 2018). In addition, multiparental populations were proven to successfully identify QTL associated with physiologically related senescence genes (Camargo et al., 2016). One important aspect is that late senescence allows a longer time in the field and shows agronomic advantages, accumulates more dry matter during the grain filling, and leads to higher yields (Valentinuz and Tollenaar, 2004). In turn, the grain filling depends on two sources such as carbon and nitrogen: the first one is provided by the transfer of photo assimilates directly to the grain from the photosynthetically active leaves, and the second one is supplied by the redistribution of photo assimilates stored in the reserve tissues before or after flowering (Yang and Zhang, 2006). Therefore, high-throughput screening of physiological features serves as proxy for QTL identification of biochemical changes during leaf aging.

Leaf senescence is a highly complex process that requires the expression of specific genes (Penfold and Buchanan-Wollaston, 2014) during aging. Genes whose transcript expression are upregulated during senescence are referred as senescence-associated genes “SAGs” and have been extensively studied and identified (Weaver et al., 1998; Munaiz et al., 2020). Senescence-associated genes functional diversity studies suggests that leaf senescence is programmed involving various cellular events, including degradation processes (Quirino et al., 2000), chlorophyll degradation, coupled with chloroplast dismantle (Sakuraba et al., 2014; Jagadish et al., 2015). However, SAGs are not only expressed during aging natural senescence but also respond to other environment cues, such as stress response and hormones, which induce early senescence (Weaver et al., 1998). Senescence-associated gene genetic studies used dark incubation treatment to induce leaf senescence. Senescence-associated genes expressed under this treatment condition were reported as primarily associated to stress conditions (Kleber-Janke and Krupinska, 1997; Park et al., 1998) and were not as highly expressed during natural senescence (Becker and Apel, 1993; Weaver et al., 1998). On the other hand, other SAGs have been identified as primarily expressed during natural senescence and were not highly expressed due to other factors. One of these genes is SAG12 in *Arabidopsis*, a gene that encodes a cysteine protease influenced by auxin, cytokinin, and sugars. SAG12 homologous in barley (*Hordeum vulgare*), the HvS40 gene, has been used as a molecular marker of age-mediated leaf senescence because its expression inversely correlates with the photosynthetic efficiency decline in barley (Humbeck et al., 1996; Jehanzeb et al., 2017).

In this study, we analyzed phenotypic profiles derived from senescence physiological trait screening of a large, eight-founder, “MAGIC” maize population to evaluate the genetics underlying the temporal changes in key developmental traits such as chlorophyl, PII quenching, and fluorescence. Time differences at silking and 2 months later were measured and were mapped to the maize genome. Molecular markers were identified, and the transcript expression of candidate genes were individually assessed in a diverse panel of genotypes. We demonstrated that physiological traits helped to identify crucial putative markers that could not be captured otherwise. The candidate gene expression analysis approach provided further genetic evidence of the role of this gene during the plant crop development, and it may serve as an important gene for breeding senescence-related genotypes.

## Materials and Methods

### Plant Material

The parents of the MAGIC population belong to the no stiff stalk heterotic group and most of them derived directly from different European landraces. This multiparent population was developed as described in Butron et al. (2019) and Jiménez-Galindo et al. (2019), and it is formed of 672 recombinant inbred lines (RILS) over six cycles of recombination in an eight-way cross synthetic. The diverse genetic base and the significant number of RILs allows for a high resolution of putative QTL-associated regions as shown in other studies, for example, corn borer and fusarium ear rot resistance (Butron et al., 2019; Jiménez-Galindo et al., 2019), forage digestibility (Lopez-Malvar et al., 2021), maize stover yield and saccharification efficiency (López-Malvar et al., 2021), and early development in cold conditions (Yi et al., 2020).

### Experimental Design and Trait Evaluation

In 2014 and 2015, a subset of the 672 RILs and the 8 founder inbred lines were evaluated at Pontevedra, Spain. The experiments were arranged in a modified augmented design with 16 blocks and 50 lines within each block, 42 RILs, and 8 testers. Each experimental plot was composed of 17 plants and a total of 2.45 m^2^. Two seeds were sown per position at 0.18 m between plants and 0.80 m between rows. Plantlets were manually thinned to a single plant per position obtaining a final density at 70,000 plants ha^–1^. Standard agronomical practices were carried out. Physiological traits were measured from the middle part of each ear leaf at two time points, silking stage (_1) and two months later (_2): chlorophyll index (Chlo) calculates the total chlorophyll content of a leaf, minimum chlorophyll fluorescence (F0) measures the light re-emitted by leaf chlorophyll molecules in the absence of photosynthetic light, and maximum quantum yield of photosystem II (Fv/Fm) that is a measure of the intrinsic (or maximum) efficiency of PSII. The trait measurements were taken from three plants of each plot using a chlorophyll content meter (CCM200, Opti-Sciences, Hudson, NH, United States) and chlorophyll fluorometer (OS-30p, Opti Sciences Inc., Hudson, NH, United States), respectively. The chlorophyll fluorescence was measured on leaves by the saturation pulse method, a method in which leaves are adapted in dark conditions for 20 min prior to the measurement. In addition, agronomic traits were evaluated: visual score (visual) was recorded according to a subjective visual scale of 1–5 (1 = dead leaves and 5 = completely green and healthy leaves), grain yield (t^.^ha^–1^), and flowering in days to silking.

Minimal level of fluorescence, F0, indicates the minimum energy required to excite chlorophyll and reactor centers to activate electron transport via PSII (Murchie and Lawson, 2013). The rise in F0 is usually associated with heat losses from the PSII and the inactivation of the PSII reaction centers leading to oxidative damage (reduction of Fv/Fm) (Long et al., 1994). Rate changes in Fv/Fm are due to a modification in the efficiency of non-photochemical quenching that can be seen as an indicator of plant photosynthetic performance. The plant will show photosynthesis inhibition under stress conditions when values are below 0.83, which is considered the optimum target value for most plant species (Björkman and Demmig, 1987; Johnson et al., 1993).

### DNA Isolation and Genotyped by Sequencing

Genomic DNA was isolated from 672 RILs along with their 8 parental lines. Coleoptiles were collected and used for genomic DNA extraction using DNasy Plant Minikit (Qiagen, Hilden, Germany) according to the manufacturer’s instructions. Quality and quantity of DNA was checked in a Nanodrop 2000 spectrophotometer (Thermo Scientific, Wilmington, DE, United States) and on agarose gels. The complete set, progenitor, and the 672 RILs were genotyped by sequencing (GBS) (Elshire et al., 2011) at the Biotechnological Institute of the Cornell University with a total of 955,690 single nucleotide polymorphisms (SNPs). Chromosomes and positions were located according to the version 2 of the maize reference genome B73 (Sen et al., 2010). These SNPs were filtered using the Tassel program version 5.2.40 (Bradbury et al., 2007); subsequently, polymorphic SNPs that were sequenced in at least 50% of the RIL were selected. Minor allele frequency was set at 0.05 to eliminate monomorphic SNPs or with rare alleles. In addition, SNPs with more than two alleles and deletions and insertions were eliminated. Genotypes of both RIL or founders with more than 5% of heterozygous SNP were considered as lost data in the analysis. After filtering, a database was generated with 465 RIL common lines in both locations and the seven parents (except parent EP17 due to seed quality) with a total of 224,363 SNPs, which were used in the subsequent analyzes.

### Statistical Analyses

Analysis of variance were performed in a two-way approach in SAS software 9.4 [version 9.4 (SAS Institute Inc., Cary, NC)], by environment and the two environments together to account for genotype by environment interaction. As detailed in the field experimental design section, RILs were grown in an augmented design. To calculate the variance attributable to each factor, we modeled each phenotype considering inbred lines as fixed effects, and environments and blocks as random effects. The model used was


$$y_{i\,j\,k}=\mu +g_{i}+\beta_{k}+t_{j}+g\,t_{i\,j}+\varepsilon_{i\,j\,k}$$

where μ is the overall mean, *g*_*i*_ is the fixed effect of the *i*th maize line, *β_*k*_* is the random effect of *k*th block, *t*_*j*_ is the randomized effect of the *j*th environment, *gt*_*ij*_ is the effect of the *ij*th genotype by environment, and *ε_*ijk*_* are residuals. Models were fitted in SAS 9.4 [version 9.4 (SAS Institute Inc., Cary, NC)] using mixed model procedure (PROC MIXED). Estimations of genotypic effects were recorded as the best linear unbiased estimator (BLUEs), and based on the combined analysis, heritabilities (h^2^) were estimated for each trait according to Holland et al. (2003). In addition, pairwise genetic correlations (r_*g*_) and phenotypic correlations (r_*p*_) were calculated as described in Holland (2006).

### Association Mapping

Genome-wide association analysis (GWAS) was performed in Tassel 5.2.40 (Bradbury et al., 2007) following the mixed linear model (MLM):


y=X⁢β+Z⁢γ+e

where *y* is the vector of the phenotypes (BLUEs of the RILs), *β* is a vector of fixed effects, including the SNP marker tested, *γ* is a vector of random additive effects (inbred lines), ***X*** and ***Z*** represent design matrices that relate and with *β* and ***γ***, respectively, and *e* is a random residuals vector. The variances of the random lines were modeled as:


$$V\,a\,r\,\left(\gamma \right)=\text{K}\,\sigma_{a}^{2}$$

where **K** is the matrix n × n of kinship coefficients, and $\sigma_{a}^{2}$is the estimated additive genetic variance (Yu et al., 2006). Estimates of maximum restricted likelihood of the variance components were obtained using the optimal compression level and P3D population parameters described in Zhang et al. (2010). Significantly associated SNPs were determined as the point where the observed and expected F test statistics deviated in the Q–Q plot, and the threshold was –logp > 4 (Gowda et al., 2015). To test for marker association, we used a threshold (–logp) > 4 and p < 0.05 as a cutoff value in the multiple QTL analysis.

The linkage disequilibrium (LD) measure (r^2^) was determined in the regions containing each of the SNPs significantly associated with each phenotypic trait. For each significant SNP, linkage blocks were established using the Haploview software (Barrett, 2009). Within the LD blocks (≤1 Mbp), candidate genes were identified and characterized in the MaizeGBD genome browser (Harper et al., 2011). Additive effects of each haplotype were calculated by adding the phenotypic values of the haplotypes that showed higher values for the trait and subtracting the phenotypic values of the haplotypes that showed lower values for that trait.

### Transcriptome Expression for QTL Validation

To validate the putative genes associated with the physiological traits, we used RNA-seq data described in Caicedo Villafuerte (2018). Briefly, RNA-sequencing library was prepared based on Illumina standard instruction according to TruSeq Stranded RNA LT (Illumina, San Diego, CA, United States). Quality control of the library DNA was evaluated, checking the concentration and the size distribution with the Agilent 2100 Bioanalyzer (Agilent, Santa Clara, CA, United States) to meet Illumina HiSeq 4000 PE100 platform system before the sequencing was performed. To assess read sequencing quality, we used FastQC (Andrews, 2010). Adapters contained in the reads were removed using the Cutadap program^1^ (Martin, 2011). The complementary DNA (cDNA) sequence from B73 file was used as reference, downloaded from www.maizegdb.org, derived from Maize B73 genome assembly (Sen et al., 2010), using the Bowtie 2, version 2.3.0 (Langmead and Salzberg, 2012) and Tophat, version 2.1.1 (Trapnell et al., 2009) tools. Fold change differences and p-values of differential expressed genes were calculated in R environment (version 3.6.3^2^) using EdgeR package (version: 3.28.1) (Robinson et al., 2010). The gene transcript expression was evaluated in six inbred that differed by the senescence delay (Supplementary Table 1), inbred NC292 with early senescence, the inbred PHT10 with mid-early senescence and the inbred PHW52 with middle senescence phenotypes, the inbred PHHB9 with middle senescence phenotype, the inbred PA8637 with mid-late senescence phenotype, and the inbred PHW79 with late senescence phenotype. Time sequence samples from each genotype were taken at silking, 15 days after silking, 30 days after silking, 45 days after silking, 65 days after silking, and 90 days after silking depending on senescence earliness as described in Supplementary Table 1.

## Results

### Phenotypic Variation

Analysis of traits across times indicated, in general, that among the MAGIC lines and their parents, there was a substantial variation in senescence performance. Analysis of variance for all phenotypes evaluated showed significant differences at silking stage (_1) and 2 months (_2) after silking among RILs and founders (*p* < 0.05). All traits showed a decrease between silking stage (_1) and two months after silking (_2). For visual scores, senescence showed a 2.41-fold change decrease; for chlorophyll index (Chlo), a 5.25-fold decrease; for minimum fluorescence (F0), a 1.18-fold decrease; and for the PII quenching ratio, a decline of 2.15-fold (Table 1).

**TABLE 1:** Averages values of 465 RIL and means of founder parents for three physiological traits and senescence visual scores evaluated at two phenological stages in the MAGIC population across two environments in Pontevedra, Spain.

**Genetic source**	**Physiological traits^*y*^**							
	**Silking evaluation^*z*^**				**Two months after silking evaluation^*z*^**			
	**Chlo_1**	**F_0__1**	**F_*v*_/F_*m*_ _1**	**Visual_1**	**Chlo_2**	**F_0__2**	**F_*v*_/F_*m*_ _2**	**Visual_2**
RILs								
Means	43.54 ± 0.41	63.74 ± 0.20	0.740 ± 0.001	3.74 ± 0.01	8.18 ± 0.35	54.28 ± 1.29	0.344 ± 0.010	1.55 ± 0.03
Range	6.8–74.6	33.0–96.0	0.446–0.811	2–5	0.7–56.9	0.0–230.30	0.006–0.787	1–4
Parents								
A509	38.23	60.61	0.744	3.51	1.78	18.82	0.102	1.08
EP125	31.28	62.15	0.743	3.56	2.92	40.18	0.268	1.19
EP43	41.33	83.67	0.679	3.79	5.88	44.11	0.160	1.13
EP53	62.27	64.75	0.746	3.70	10.04	57.73	0.366	1.52
EP86	38.07	63.78	0.738	3.97	7.21	48.34	0.375	1.69
F473	53.45	67.01	0.729	3.72	4.64	80.35	0.418	1.37
PB130	44.19	57.86	0.755	3.60	8.85	61.32	0.539	1.50
EP17	–	–	–	–	–	–	–	–
LSD (*p* > 0.05)	8.30	1.74	0.02	0.54	8.17	36.16	0.27	0.39

*^y^Physiological traits where Chlo is chlorophyll index, F_0_ is minimum fluorescence, F_v_/F_m_ is maximum quantum yield of PSII, and Visual is visual aspect with scale 1 (senescence) to 5 (green).*

*^z^Subindex followed by _1 indicates that the trait was evaluated at silking point, and that followed by _2 means that it was evaluated 2 months after silking.*

Founder parents of the MAGIC population also showed significant differences among them (*p* < 0.05) for all traits. For visual assessment, founder lines had a score above 3 units at silking stage, whereas the scoring 2 months later was significantly lower at ∼1 (Table 1). All other physiological values changed in ranking between the silking stage and the second measurement 2 months after silking, indicating the phenotypic variability of the parental lines of the MAGIC population. For example, Chlo ranged from 31.23 to 62.27 at silking, while 2 months later, this range was much lower, between 1.78 and 10.04. For the trait F_0_, founders at silking stage had a minimum value at 57.86 and a maximum at 83.67, whereas 2 months after silking, F_0__2 ranged from 18.82 to 80.35. For PSII quenching, the values observed across all founder parents were at 0.740, except for EP43 that had the lowest rate at 0.679. However, 2 months after silking, F_*v*_/F_*m*__2, the parent F473 had the highest value at 0.539.

Recombinant inbred lines (RILs) had a large phenotypic variability in both scoring times. At silking evaluation, for visual scoring, inbreds showed a phenotypic range between 2 and 5; Chlo range was between 6.8 and 74.6; F_0_ ranged from 33.0 to 96.0; and F_*v*_/F_*m*_ had a variation ranging from 0.446 to 0.811 units. At scoring time, 2 months after silking, visual phenotypes ranged from 1 to 4; Chlo phenotypic variation was between 0.7 and 56.9 units; F_0_ ranged from 0.0 to 230.0; and for F_*v*_/F_*m*_, the phenotypic variation ranged from 0.006 to 0.787 units (Table 1).

### Heritability and Correlation Analysis

Heritability was high for all the physiological traits. F_0__1 had the highest value at 0.81 followed by Chlo_1 at 0.75 and F_*v*_/F_*m*_ _1 that had a 0.58 value. Heritability values were slightly lower for these traits when measured 2 months after silking at 0.63, 0.49, and 0.42, for Chlo_2, F_*v*_/F_*m*_ _2, and F_0__2, respectively. However, visual aspect evaluated at silking point had the lowest heritability at 0.27, and increased substantially after 2 months with a 0.69 value for visual_2 (Table 2, above diagonal). We calculated pairwise genetic correlations among the four traits evaluated at two different time points obtaining 11 out of 28 correlations among pair of traits significantly correlated with *r* > |0.70|. Senescence visual assessment at silking stage (visual_1) showed positive relationships *r* > 0.62 within five out of eight pairwise comparisons. In addition, minimum chlorophyll fluorescence (F_0__2) evaluated 2 months after silking was significantly correlated with six out of eight of the other traits compared, with correlation coefficients at *r* > 0.23 for the trait F_0__1 to a correlation *r* at 1.00 for visual_1 (Table 2). As for the phenotypic correlations, only two pairwise comparison had *r* > 0.60, visual_2 with Chlo_2 and Fv/Fm_2, while the remaining correlation coefficients were relatively low varying between 0.08 and 0.55 (Table 2, below diagonal). Among all the phenotypic correlations Fv/Fm_2–Visual_2 was the highest at 0.7.

**TABLE 2:** Genotypic and phenotypic correlation coefficient matrix of physiological traits and senescence visual appearance evaluated in the MAGIC population EPS21 across two environments in Pontevedra, Spain.

**Phenotype^*x*^,^*w*^**	**Chlo_1^*z*^**	**F_0__1**	**F_*v*_/F_*m*__1**	**Visual_1**	**Chlo_2^*z*^**	**F_0__2**	**F_*v*_/F_*m*__2**	**Visual_2**
Chlo_1		–0.01	0.36^*^	0.70^*^	0.43^*^	0.32^*^	0,30^*^	0,17^*^
F_0__1	–0.08		–0.61	–0.03	0.05	0.23^*^	–0.03	–0.03
F_*v*_/F_*m*__1	0.26^*^	–0.47		0.62^*^	0.26^*^	0.17	0.27^*^	0.29^*^
Visual_1	0.20^*^	0.01	0.13^*^		0.93	1.00^*^	0.96^*^	0.76^*^
Chlo_2	0.26^*^	–0.01	0.13^*^	0.20		0.75^*^	0.87^*^	0.83
F_0__2	0.14^*^	0.12^*^	0.08^*^	0.24^*^	0.47^*^		0.85^*^	0.89^*^
F_*v*_/F_*m*__2	0.10^*^	0.02	0.10^*^	0.19^*^	0.55^*^	0.83^*^		1.00^*^
Visual_2	0.09^*^	–0.02	0.14^*^	0.24^*^	0.64	0.51^*^	0.60^*^	
*h* ^2^	0.75	0.81	0.58	0.27	0.63	0.42	0.49	0.69
*Flow* ^*y*^	–0.15	0.12	–0.01	–0.31	0.15	0.36	0.31	0.40
*Yield*	0.18	–0.16	0.20	0.48	0.24	0.13	0.15	0.23

*Below the line, heritabilities (h^2^) and Pearson correlations with flowering time (flow) and yield.*

*^x^Physiological traits where Chlo is chlorophyll index, F_0_ is minimum fluorescence, F_v_/F_m_ is maximum quantum yield of PSII. and Visual is visual aspect with scale 1 (senescence) to 5 (green).*

*^y^Flow is flowering time at silking measured in days after sowing, and yield was measured in tons per hectare (t/ha).*

*^w^Genetic correlations above the diagonal (r_g_) and phenotypic correlations below the diagonal (r_p_).*

*^z^Subindex followed by 1 and 2 indicates that the trait was evaluated at silking point or evaluated 2 months after silking, respectively.*

^*^Correlation coefficient exceeded two times their standard error.

Pearson’s correlations of flowering time with the physiological traits at silking time point varied from –0.31 to 0.12; visual_1 had the lowest correlation at –0.31, and the trait F0_1 had the highest value at 0.12. Furthermore, correlations evaluated 2 months later ranged from 0.15 to 0.40, being Chlo_2 the lowest and visual_2 the highest values, respectively. For yield performance, Pearson’s correlations with the physiological traits at silking time point ranged between –0.16 and 0.48, corresponding to the traits F0_1 and visual_1, respectively. Yield evaluated 2 months after silking varied from 0.13 to 0.24, corresponding to F0_2 and Chlo_2, respectively.

### Quantitative Trait Loci

We developed a genome-wide association studies (GWAS) using the RILs population previously genotyped utilizing a GBS technology and assessed individual phenotypes at both time point, silking stage (_1) and 2 months after silking (_2) stage, to identify loci associated with all the traits. Each candidate SNP associated to the trait was then studied in Haploview to account for LD and to identify the haplotype interval linked to the trait of interest.

For visual senescence, GWAS analysis detected eight significant SNPs associated at silking stage on chromosome (chr) 2, chr3, chr4, and chr8 (Supplementary Figure 1 and Supplementary Table 2). The percentage of phenotypic variance explained for each putative marker ranged from 5.1 to 7.7%. Visual senescence studied 2 months after silking resulted in 27 SNPs localized on chr3, chr6, chr7, chr9, and chr10. For these putative QTLs, the proportion of phenotypic variance explained ranged between 4.8 and 8.8% (Supplementary Figure 2 and Supplementary Table 2). We observed that peak QTLs became highly significant haplotypes at time point, 2 months after silking (Table 3), detecting four QTL interval haplotypes. These intervals corresponded to the markers on chr3 with an interval region of 87 kbp between 88 and 89 Mb (90% confidence region), another marker on chr3 with an interval region of 7 kbp between 90.3 and 90.4 Mb, the marker on chr6 with the interval region of 471 kbp at 56–57 Mb, and the QTL at marker on chr10, S10_2034863 (Table 3).

**TABLE 3:** QTLs mapped with significant SNPs associated across traits and evaluation time point.

**Trait**	**Time**	**SNP**	**Flanking markers**	**A509**	**EP125**	**EP17**	**EP43**	**EP53**	**EP86**	**F473**	**PB130**	**TQTL**	**ΣEAT**	**ΣEAR**	**EAP**
Visual	2MAS	S3_8974495	S3_8888862–S3_8976187	–0.8	–0.1, –0.2	0.4, –0.2	–0.1, –0.7	–0.1, –0.6	0.5, 0.2	0.4, –0.7	0.2, –0.7	87	1.4	0.8	0.1
Visual	2MAS	S3_9038739	S3_9035419–S3_9042531	0.04, 0.02	–0.1	0.2, –0.1	0.1, –0.2	–0.1, –0.2	0.6, 0.1	0.3, –0.1	0.4, –0.1	7	0.9	0.7	0.3
Visual	2MAS	S6_56810498	S6_56809291–S6_57280494	–0.4	–0.3, –0.4	–0.3, –0.4	–0.4	0.3, 0.2	–0.4	0.3, 0	–0.1, –0.3	471	0.7	0.2	0.0
Visual	2MAS	S10_2034863	S10_2034863–S10_2034863	0.0	0.0	0.2, 0	0.2	0.2, 0	0.0	0.0	0.0	0	0.2	0.2	0.2
Chlorophyl	Silking	S1_278163516	S1_278163476–S1_278352686	–10.7, –19.4	–16.5, –19.4	–8.2, –11	–3.6, –8	6.5, 2.8	3.1, –5.1	6.5	6.5	189	32.4	11.6	5.3
Chlorophyl	Silking	S1_278356935	S1_278353505–S1_278357906	3.5, –5.3	-2.8	7.9, 4.6	3.1, –3	7.9, 2.3	7.9, –3.9	7.2	7.2	4	13.3	9.5	0.3
Chlorophyl	Silking	S1_278992401	S1_278909244–S1_279068377	–1.6, –3.8	–4.5	–2.2, –4.5	–3.8	–2.4	–3.8, –4.5	–0.2	1.4	159	5.9	1.9	1.9
Chlorophyl	2MAS	S1_279901234	S1_279901234–S1_279901243	4.5	0.0	0.0	4.5	0.0	0.0	4.5	4.5	0.009	4.5	2.3	2.3
Chlorophyl	2MAS	S1_279901243	S1_279901234–S1_279901243	4.5	0.0	0.0	4.5	0.0	0.0	4.5	4.5	0.009	4.5	2.3	2.3
Chlorophyl	2MAS	S3_201194856	S3_201189615–S3_201228263	–2.6, –7	–5.9, –11.1	12.4, –7.5	1, –0.1	–2.1, –6.6	11.1, 5	–6.2, –11.1	–6.3	39	24.6	3.3	4.0
Chlorophyl	2MAS	S3_201538092	S3_201538092–S3_201545231	–19.1	–19.1	1.9, –19.1	1.9, –1.6	–19.1	–1.6, –3.5	–19.1	–19.1	7	10.5	1.5	1.5
Chlorophyl	2MAS	S3_201538103	S3_201538092–S3_201545231	–19.1	–19.1	1.9, –19.2	1.9, –1.7	–18.1	–1.6, –3.6	–19.1	–19.1	7	10.5	1.5	1.5
Chlorophyl	2MAS	S3_201538104	S3_201538092–S3_201545231	–19.1	–19.1	1.9, –19.3	1.9, –1.8	–17.1	–1.6, –3.7	–19.1	–19.1	7	10.5	1.5	1.5
Chlorophyl	2MAS	S3_201538113	S3_201538092–S3_201545231	–19.1	–19.1	1.9, –19.4	1.9, –1.9	–16.1	–1.6, –3.8	–19.1	–19.1	7	10.5	1.5	1.5
PSII Quenching	2MAS	S3_207022252	S3_207022212–S3_207022252	171.1, 11.1	11.1	171.1, 0	0.0	171.1, 11.1	171.1, 0	11.1	11.1	0.04	85.5	49.5	49.5

*Linkage blocks with flanking markers, QTL length, haplotype that increases or decreases the effect, sum of the real and theoretical additive effect of the favorable haplotype, and additive effect of the parents for each SNP or SNPs group significantly associated with Chlo_1, Chlo_2, and Fv/Fm_2, evaluated in the EPS21 MAGIC population, in Pontevedra, Spain.*

*TQTL, QTL size in kbp; HF, haplotype that increases the additive effect; HD, haplotype that reduces the additive effect; ΣEAT, sum of the theoretical additive effect, resulting from the sum of the additive effects of all significant SNPs in the linkage block; ΣEAR, sum of the real additive effect, resulting from the sum of the additive effects of all significant SNPs in the linkage block and subtracted from the total average of the entire population; EAP, additive effect based on the genotype of the parental.*

For chlorophyll, we detected 32 SNPs at silking stage, distributed across all chromosomes, except chr2, chr6, and chr9 (Supplementary Figure 1 and Supplementary Table 3). The percentage of phenotypic variance explained by a single SNP ranged from 4.0 to 8.3%, on average. The total number of unfavorable alleles detected across founder lines was twice as much of the favorable alleles: for instance, EP125, the founder with the lowest chlorophyll index at silking stage, had the highest number of unfavorable alleles (22) across all the parental lines. At time point 2 months after silking, 19 SNPs were detected, and they were located on chr1, chr3, chr4, chr5, and chr6 (Supplementary Figure 2 and Supplementary Table 3). The percentage of phenotypic variance explained by a single SNP was, on average, slightly higher than at silking stage ranging between 4.7 and 8.7%. As expected, the favorable alleles positively controlling chlorophyl decreased as plant senescence developed resulting in ∼1/4 of the total alleles (21/85). After studying the linkage blocks for each of these putative significant SNPs, we identified three QTL interval haplotypes on chr1 at silking stage, and three QTL haplotypes when evaluation took place 2 months after silking, namely, two QTLs on chr1 and one QTL on chr3, altogether resulting in seven of the significant putative markers (Table 3). The QTL region on chr1 was significant at both evaluation times. However, chr3 was detected only at 2 months after silking evaluation time. On chr3, the significant SNP (*p* = 3.86E–6) was located at 201,538,092 bp explaining 5.3% of the phenotypic variation (Supplementary Figure 2B). These findings suggest that chr3 region may have more importance at late stages of plant development as the plant matures.

For PSII quenching, 19 SNPs were detected to be significantly associated at silking stage evaluation. There were six putative markers on chr1, in addition to one marker on each chr4, chr6, and chr10, respectively (Supplementary Figure 1 and Supplementary Table 4). On average, the percentage of the phenotypic variance explained by a single SNP ranged from 4.2 to 8.3%, and the additive allelic substitution effect fluctuated between 4.97 and 10.16 units. The Manhattan plot depicted a high peak on chr1 (Supplementary Figure 1C) with the most significant SNP at position 47,775,156 bp (*p* = 9.35E–6) explaining 5.4% of the phenotypic variance. Quantitative trait loci analysis of PSII quenching evaluated 2 months after silking detected two SNPs significantly associated, one SNP located on chr3 and another one on chr6 (Supplementary Figure 2 and Supplementary Table 4). The phenotypic variance explained by a single SNP was, on average, lower than the earlier stage, ranging between 5.4 and 5.5%. However, additive effects at loci were much higher than at silking point with 79.98 and 103.79 units (Supplementary Table 4). For the 2 months after silking measurement, we identified a QTL associated to a haplotype on the chromosomal region of chr3. This allelic region corresponding to marker S3_207022252 had an additive effect at 85.5, and it was located within an interval region of 0.04 kbp flanking markers at 20.7 Mb position (Table 3). This QTL was not significant at silking stage but was highly significant at later maturity of the plant development.

For fluorescence (F0), QTL analysis detected 43 SNPs significantly associated to chlorophyl fluorescence at silking stage evaluation. Among these putative QTL markers, 35 SNPs were found on chr1, 2 SNPs were detected on chr2, 3 SNPs on chr3, and 1 putative marker on chr5, ch6, and chr7, respectively (Supplementary Figure 1 and Supplementary Table 5). On average, the percentage of phenotypic variation explained by a single SNP ranged between 0.043 and 0.108. The Manhattan plot depicted a high peak on chr1 (Supplementary Figure 1D) with the most significant SNP at position 26,037,654 bp (*p* = 8.44E–07) explaining 6.8% of the phenotypic variance. Fluorescence measured at 2 months after silking, we reported 20 putative markers significantly associated with this trait: 18 SNPs located on chr3, 1 marker on chr 5, and another 1 on chr8 (Supplementary Figure 2). On average, the percentage of the phenotypic variance explained by a single SNP ranged from 4.6 to 8.45%, and the additive effect ranged between 6.82 and 14.61, on average (Supplementary Table 5). The QTL peak had a significant SNP on chr3 located at 201,538,222 bp (*p* = 2.02E–6) explaining 6.3% of the phenotypic variation (Supplementary Figure 2D).

### Candidate Genes and Expression Analysis

We used the putative QTLs significantly associated with all the traits to search for candidate genes annotated developing a BLAST search on the maize genome browser. These searches resulted in candidate genes for all the phenotypic traits studied. For visual senescence evaluated at matured stage (Visual_2), there were seven candidates on chr3. For chlorophyl, there were three candidate genes at silking stage (Chlo_1) located on chr1 and five candidate genes on chr3 that were associated with the trait measured 2 months after silking (Chlo_2). Finally, for PSII quenching, we observed two candidate genes associated with this trait at late plant maturity (F_*v*_/F_*m*__2) (Table 4).

**TABLE 4:** Candidate genes and physical positions for each significant SNP associated with Chlo_1, Chlo_2, and F_*v*_/F_*m*__2, visual senescence, and their respective descriptions.

**Chr^*a*^**	**Trait**	**SNP physical position (bp)**	**Gene ID and position (B73 RefGen_v2)**	**TAIR database description**
1	Chlo_1	278,163,516	Zm00001d034071 (S1_278113793–S1_278126352)	Nuclear-encoded chloroplast stromal cyclophilin CYP20-3 (also known as ROC4). Protein is tyrosine phosphorylated, and its phosphorylation state is modulated in response to ABA in *Arabidopsis thaliana* seeds.
1	Chlo_1	278,356,935	Zm00001d034076 (S1_278418921–S1_278425448)	Cyclic nucleotide-regulated ion channel family. “defense, no death” gene (DND1) encodes a mutated cyclic nucleotide-gated cation channel; Same as CNGC2 (article ID 229): Cyclic nucleotide gated channel, activated by cAMP, conducts K^+^ and other monovalent cations but excludes Na^+^. Conducts Ca^2+^ into cells, which is linked to the generation of NO and the NO signaling pathway involved in the innate immune response to pathogens. CNGC2 could be the key step mediating bulk Ca^2+^ influx into leaf cells after unloading from the vascular and have no direct roles in the leaf development and HR.
1	Chlo_1	278,992,401	Zm00001d034099 (S1_278996225–S1_278999894)	Encodes a leaf-type ferredoxin: NADP(H) oxidoreductase.
1	Chlo_2	279,901,234	Zm00001d034137 (S1_279742698–S1_279744047)	Encodes a chloroplast localized subunit of casien kinase4, and the casein kinase II. CK2-mediated phosphorylation enhances the light-induced degradation of PIF1 to promote photomorphogenesis.
1	Chlo_2	279,901,243	GRMZM2G003421 (S1_279824465–S1_279826154)	Encodes a member of F-box proteins that includes two other proteins in Arabidopsis (ZTL and FKF1). Overexpression results in arrhythmic phenotypes for a number of circadian clock outputs in both constant light and constant darkness, long hypocotyls under multiple fluences of both red and blue light, and a loss of photoperiodic control of flowering time.
3	Chlo_2	201,194,856	Zm00001d043574 (S3_ 201165018–S3_201167249)	Chloroplastic NifS-like protein that can catalyze the conversion of cysteine into alanine and elemental sulfur [S (0)] and of selenocysteine into alanine and elemental Se [Se (0)].
3	Chlo_2	201,538,092	Zm00001d043586 (S3_ 201322694–S3_201323761)	Senescence regulator (protein of unknown function, DUF584)
3	Chlo_2	201,538,103	Zm00001d043589 (S3_ 201371185–S3_201375806)	MADS box gene negatively regulated by APETALA1
3	Chlo_2	201,538,104	Zm00001d043607 (S3_ 201861549–S3_201871591)	Encodes a hexokinase (HXK1) in the plant glucose-signaling network. Functions as a glucose sensor to interrelate nutrient, light, and hormone signaling networks for controlling growth and development in response to the changing environment
3	Chlo_2	201,538,113	Zm00001d043609 (S3_ 201966295–S3_201973589)	MAP kinase kinase 6. Encodes a member of the MAP Kinase Kinase family of proteins. It can phosphorylate MPK12 *in vitro*, and it can be dephosphorylated by MKP2 *in vitro* (ANQ1, ATMKK6, MKK6).
3	F_*v*_/F_*m*__2	207,022,252	Zm00001d043800 (S3_ 207029164–S3_207037370)	Serine/threonine kinase family catalytic domain protein
3	F_*v*_/F_*m*__2	207,022,252	Zm00001d043801 (S3_ 207037486–S3_207041394)	Alpha/beta-hydrolases superfamily protein
3	Visual_2	8,974,495	Zm00001d039566 (S3_ 8887555–S3_8888747)	(HSP17.6II) 17.6 kDa class II heat shock protein
3	Visual_2	8,974,495	Zm00001d039575 (S3_ 8980485–S3_8982638)	A single copy gene that encodes a protein with sequence similarity to tomato E8 (ACC oxidase, the last step in ethylene biosynthesis) involved in ethylene synthesis and fruit ripening in tomato. This gene is not induced by ethylene in siliques. The transcript is found in siliques, etiolated seedlings, leaves, stems, and flowers.
3	Visual_2	9,038,739	Zm00001d039578 (S3_9037860–S3_9042655)	Histidyl-tRNA synthetase, key player in translation and act early in protein synthesis by mediating the attachment of amino acids to their cognate tRNA molecules
3	Visual_2	9038,739	Zm00001d039579 (S3_ 9045175–S3_9047443)	phd34—PHD-transcription factor 34, RING/FYVE/PHD zinc finger superfamily protein
3	Visual_2	9,038,739	Zm00001d039581 (S3_9072834–S3_9073771)	ca5p15—CCAAT-HAP5-transcription factor 515, nuclear factor Y, subunit C13
6	Visual_2	56,810,498	GRMZM2G306677 (S6_56815895–S6_56820763)	A member of ARF GAP domain (AGD), *A. thaliana* has 15 members, grouped into four classes.
10	Visual_2	2,034,863	Zm00001d023261 (S10_2008922–S10_2010657)	Nucleotide-sugar transporter family protein
10	Visual_2	2,034,863	Zm00001d023262 (S10_2011814–S10_2017183)	brk3—brick3, (GRL, NAP1, NAPP) transcription activators
10	Visual_2	2,034,863	Zm00001d023263 (S10_2039154–S10_2041723)	Glutamine-fructose-6-phosphate transaminase that likely plays a role in UDP-N-acetylglucosamine biosynthesis.

*^a^Chr, chromosome; Chlo_1 and Chlo_2 = chlorophyll index evaluated at silking and two months after silking; F_*v*_/F_*m*__2 = maximum quantum yield of photosystem II evaluated 2 months after silking; and Visual_2 = visual scoring senescence evaluated 2 months after silking.*

**FIGURE 1:**
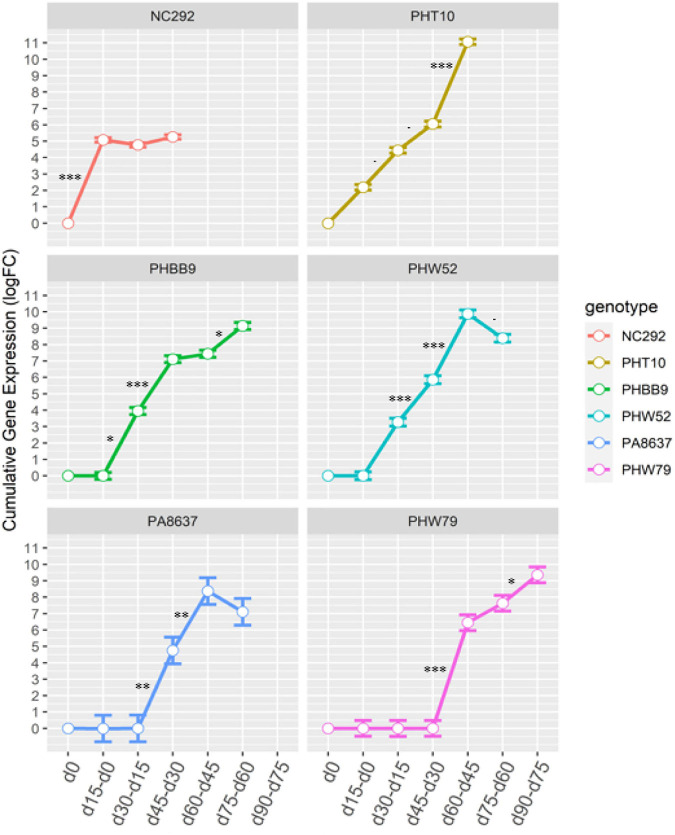
Cumulative fold-change transcript expression of SNP “Zm00001d043586” at seven time points measured in six genotypes NC292, PHT10, PHHB9, PHW52, PA8637, and PHW79 evaluated in Pontevedra, Spain. Error bars represent the tagwise dispersion of the SNP for each genotype. ^*z*^Denoted as d0 = flowering time; d15–d0 = expression between d15 and d0; d30–d15 = expression between d30 and d15; d45–d30 = expression between d45 and d30; d60–d45 = expression between d60 and d45; d75–d60 = expression between d75 and d60; d90–d75 = expression days after flowering.⋅^∗^, ^∗∗^, ^∗∗∗^ denotes significance at *p* = 0.10, *p* = 0.05, *p* = 0.01, *p* = 0.001, respectively.

The comparative genomics search resulted in a candidate gene associated with Chlo_2 (Zm00001d043586) that was annotated as an *Arabidopsis* homologous; this gene encodes for a protein involved in senescence regulation of the S40 family. To evaluate how plant aging affects the gene expression of Zm00001d043586, located on chromosome 3 at position 201,538,092 bp, we compared the transcript expression obtained from RNA-seq at different time points after silking on seven different genotypes. Time sequence samples from each genotype were measured at silking (d0), 15 days after silking (d15), 30 days after silking (d30), 45 days after silking (d45), 65 days after silking (d65), and 90 days after silking (d90) depending on senescence earliness as described in Supplementary Table 1. First, we compared gene expression between each time point relative to d0 (Table 5). Early senescence line NC292 showed highly significant differences at time point d15 with a log-fold change (FC) at 5.08 (*p* = 1.91E–14). Mid-early phenotypes such as PHT10 started to be significantly different at d30 with a logFC at 4.45 (2.22E–03). Mid-late phenotype, PA8637, showed gene expression difference to silking time at d45 with a logFC 4.76 (*p* = 3.25E–02). Finally, late senescence phenotype, PHW79, had a significant gene expression at 60 days after silking (d60) with a logFC 6.45 (*p* = 1.33E–04). Second, we studied the transcription differences between pairwise time sequence for each genotype (Figure 1). Remarkably, gene expression in NC292 was significantly different between d0 and d15, and remained not significant between d15 and d30, and d30 and d45. Mid-early and middle senescence phenotypes, such as PHT10, PHW79, and PHHB9, increased steadily the gene expression. However, genotypes PHW52 and PA8637 increased their expression later at d30 and declined after the d65 stage (Figure 1).

**TABLE 5:**
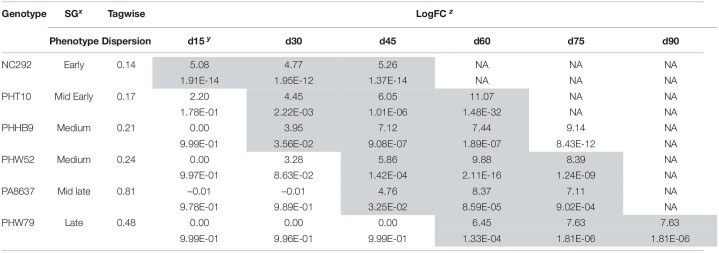
Expression profiles fold change and p-value significant differences of six divergent stay-green genotypes studied for the GRMZM2G311347 gene at seven different time points in days (d) after the compared flowering time (d0) as references, d15, d30, d45, d60, d75, and d90 evaluated in Galicia, Spain.

*Shaded values represent significant fold change expression compare to d0, flowering time.*

*^x^Visual phenotypes representing early, mid early, medium mid late and late senescence.*

*^y^Denoted as d0 = flowering time, d15 = 15 days after flowering, d30 = 30 days after flowering, d45 = 45 days after flowering, d60 = 60 days after flowering, d75 = 75 days after flowering, and d90 = 90 days after flowering.*

*^z^LogFC denotes log-fold change expression with p-values underneath each value having references d0.*

## Discussion

This research aimed to understand the genetic regions influencing senescence traits and the temporal changes between two key phenotypic stages silking (_1) and 2 months after silking (_2). We studied senescence in key physiological traits associated with plant aging: Chlo, calculates the total chlorophyll content of a leaf, F_0_ measures the minimum chlorophyll fluorescence, and Fv/Fm that represents the maximum quantum yield of photosystem II. This strategy resulted in the detection of QTLs linked with novel markers, suggesting that physiological screenings should be further investigated in the future.

Visual phenotypic correlations were low, indicating that selections based on visual scores may not be effective. Pairwise genetic correlation between all traits identified high positive (≥0.60) correlations between Chlo_1, Fv/Fm_1, and visual_1, and chlo_2, F_0__2, Fv/Fm_2, and visual_2. For example, in maize, Yang et al. (2017) reported phenotypic correlations at 0.77 for Fv/Fm and visual scores. Other studies in sorghum obtained lower correlations at 0.15 (Sukumaran et al., 2016), perhaps influenced by the difference in plant morphology of these two species or the conditions of drought stress of the sorghum experiments. However, Sukumaran et al. obtained similar visual scoring range at flowering times between 1.3 and 4.6. In our study, there was also a negative correlation between F_0__1 and Fv/Fm_1, indicating that the light exposure at early stage and its efficiency may not be sufficient at early stages of flowering time. However, that was not the case at later development, 2 months after flowering, with a high positive correlation at 0.85, suggesting that the source-sink apparatus may be more efficient toward the grain filling.

Heritability results showed that F_0__1 had the highest value at 0.81, followed by Chlo_1 and Chlo_2, at 0.75 and 0.62, respectively. This results are in agreement with those obtained by Yang et al. (2017) at 0.66 and are higher than the ones observed in Cai et al. (2012), between 0.36 and 0.57. These differences may be due to the phenotypic diversity of the founder parents used in this work whose chlorophyll content did not differ more than 57.3 points. However, Yang et al. (2017) included founder female parent “Zheng58” with functional stay green phenotypes and still observed the similar range of values as in this research. This high F0 heritability would allow for selections with reduce minimum energy to excite chlorophyll and reactor centers with sufficient intensity to induce electron transport through PSII, a desirable trait in maize germplasm. Genetic correlations were high at early stage between Fv/Fm_1 with F0_1 (0.61) and moderate with Chlo_1 (0.36). Similarly, genetic correlations were high between the physiological 2 months after silking, with values at 0.73 and 0.87 for combinations F0_2 with Chlo_2 and Chlo_2 with Fv/Fm, respectively. Because of the high heritabilities and the genetic correlations, this would suggest that Chlo, F0, and Fv/Fm would be a reliable phenotypic technique to assess plant senescence. F0_1 would be of particular importance due to its negative Pearson correlation with yield (–0.15) and the possibility of evaluation at early stage of the plant cycle. In the association analysis, we identified a total of 227 individual associated SNPs for all the evaluated traits (data not shown); however, among all these detected SNPs, based on the number of homozygous lines for a given variant, the selection of QTLs should be carefully made, providing that these observations are below 58 (465 RIL/8 parent lines = 58 lines) and that could generate false positive results due to the unequal composition of the data and the reduce accuracy, suggesting that natural selection has probably existed. Therefore, the total QTL identified for each trait was smaller because two or more of these significant SNPs can form a single linkage block but only one QTL. For instance, there were two QTLs on chr1 in the traits PSII_1 and F0_1, at silking stage, but the candidate SNPs are located at 47,775,156 bp and 26,037,654 bp, respectively, which suggests that they are different haplotypes (Supplementary Figure 1). There was no common significant QTL (SNPs) for traits evaluated at both time points, silking and 2 months later, suggesting that each QTL was specific to the plant development time point at silking and senescence stages. However, the same QTL peak was detected for traits F0_2 and Chlo_2 on the chromosome 3 genomic region at approximately 201,538,092 bp, the same region as the Zm00001d043586 candidate gene, once more suggesting the importance of this region at late developmental stages.

Our research provided novel QTLs that have not been previously reported. These molecular markers were primarily found with a flint European origin, likely because it was the first time that senescence physiological traits were studied using this genetic background. We found QTLs associated with Chlo located on chromosomes 1, 3, 4, 5, 6, 7, 8, and 10, both at silking and 2 months after silking. Similarly, Yang et al. (2017) reported six QTL on chromosomes 1, 4, 6, 8, and 9 associated with this same trait. For the Fv/Fm trait, we detected associated QTLs positioned on chromosomes 1, 3, 4, 6, and 10, whereas other researchers found nine QTLs on chromosomes 1, 2, 3, 4, 5, 6, and 8 (Yang et al., 2017).

We found two genes associated with chlorophyll index (Chlo), represented by the SNPs on chromosome 1 significantly associated with Chlo_1 (S1_278163516, S1_278356935, and S1_278992401) located within the candidate gene Zm00001d034073 in the version 2 of the B73 genome and corresponding to the gene Zm00001eb057820 in the version 5. This gene is homologous to the WRKY DNA-BINDING PROTEIN 57 gene (AT1G69310) in *Arabidopsis*, playing a role as a convergence point between jasmonic acid and auxin-mediated signaling during jasmonic acid-induced foliar senescence (Jiang et al., 2014). Although the SNP associated with this gene was only significantly associated with chlorophyll content at silking stage, and not at senescence stage, it was upregulated at later development based on the gene expression profiles studied in the seven maize inbred lines, confirming that Zm00001d034073 plays a role during naturally occurring leaf senescence.

On the other hand, SNPs significantly associated with Chlo_2 on chromosome 3 (S3_201538092, S3_201538103, S3_201538104, and S3_201538113) were located within the candidate gene Zm00001d043586. The homologous gene in *Arabidopsis* AT1G29640.1, also known as AtS40-1 (Fischer-Kilbienski et al., 2010), has been described as a senescence regulator. Proteins of the S40 family are induced during natural senescence and may also be regulated in response to hormone regulation (Krupinska et al., 2002; Zheng et al., 2019) because some proteins may only be expressed during dark-induced senescence (Kleber-Janke and Krupinska, 1997). For example, the SAG12 encoding protein identified in barley (*Hordeum vulgare*) produced by the nucleus gene HvS40 was associated with the degeneration of chloroplasts occurring during naturally occurred senescence (Krupinska et al., 2002). In our research, based on the gene Zm00001d043586 expression profiles studied in seven maize inbred lines, we detected that transcript expression was upregulated during senescence in six out of the seven lines studied when taking flowering time expression as reference. This suggests that the overall importance of this gene was due to natural induced senescence (Table 5 and Figure 1). However, we observed that the pace of the senescence response regarding to the chlorophyll degradation showed clear differences based on the RNA-seq analysis. Early senescence lines showed high levels of expression from silking stage up to d45 (Table 5); however, for the middle senescence lines, the upregulation was initiated at d45 until d75, and for the late-senescence line, PHW79, transcription started at d60 to the end of the cycle, d90 (Figure 1). Thus, our results suggest that the gene Zm00001d043586 is associated with the speed of chlorophyll dismantle at late plant cycle stages. This may be due to a difference in the chloroplast degradation (Krupinska et al., 2002). Our results also suggest that this chloroplast degradation is germplasm dependent, depending upon the type of the diversity of the genotypes, because these changes happened at later stages of plant development within the different material. The general model of senescence proposed that the initial decline rates of photosynthesis may be the senescence-initiating factor (Hensel et al., 1993), and this is not the case of the SAG12 gene because it does not rapidly respond to senescence-promoting stresses, and it is only expressed in the leaves as they age (Weaver et al., 1998). Thus, we observed a similarly trend in maize represented in Figure 1, as reported by Noh and Amasino (1999) in *Arabidopsis* and by Zheng et al. (2019) in rice.

This study provides a systematic phenological characterization of a maize MAGIC population on two temporal time points conditions. We were able to identify not previously detected QTL loci for all the traits studied related to the onset of senescence at flowering time and also at late developmental stage, 2 months later. By using a multifounder maize population, we were able to capture much of the genetic variation present in elite cultivars and in particular the flint European genetic background. Thus, fine-scaling genomic regions of the developmental and physiological patterns can be exploited for senescence line development. We showed that the Zm00001d043586 gene was significantly associated with the chlorophyll rate, and it was of particular importance at later plant development due to its rate of gene expression response. This research supports the use of MAGIC population for QTL mapping that can assist in pyramidal selection using complementary alleles for crop enhancement.

## Data Availability Statement

The datasets presented in this study can be found in online repositories. The name of the repository and accession number can be found below: National Center for Biotechnology Information (NCBI) BioProject, https://www.ncbi.nlm.nih.gov/bioproject/, PRJNA746402.

## Author Contributions

BO: conceptualization. MC and BO: data collection. EM, JJ, RM, MC, and BO: data analysis. EM and MC: writing original draft. EM, RM, BO, JJ, and MC: writing—review and editing. All authors have read and agreed to the published version of the manuscript.

## Conflict of Interest

The authors declare that the research was conducted in the absence of any commercial or financial relationships that could be construed as a potential conflict of interest.

## Publisher’s Note

All claims expressed in this article are solely those of the authors and do not necessarily represent those of their affiliated organizations, or those of the publisher, the editors and the reviewers. Any product that may be evaluated in this article, or claim that may be made by its manufacturer, is not guaranteed or endorsed by the publisher.

## Footnotes

1
http://cutadapt.readthedocs.io/en/stable/guide.html#illumina-truseq

2
https://www.r-project.org/
