# Supplementary material for: Precision Mapping of a Maize MAGIC Population Identified a Candidate Gene for the Senescence-Associated Physiological Traits
Source: Front Genet. 2021 Oct 4;12:716821. doi: 10.3389/fgene.2021.716821 (PMC8521056; doi:10.3389/fgene.2021.716821)
Supplement: Supplementary file 1 [file Table_1.docx]

| Genotype | Origin ^x^ | Accession | Pedigree | Phenotype ^y^ | RNAseq Time |
| --- | --- | --- | --- | --- | --- |
| NC292 | B73 | PI690391 | B73 x NC250P | Early | d0, d15, d30 and d45 |
| PHT10 | B37/PHG39 | PI601573 | B73 x G39 | Mid early | d0, d15, d30, d45 and d60 |
| PHW52 | B37/PHG39 | PI601575 | B73 x G39 | Middle | d0, d15, d30, d45, d60 and d75 |
| PHHB9 | B37/PHG39 | PI565104 | PHG86xPHW52 | Middle | d0, d15, d30, d45, d60 and d75 |
| PA8637 | Mixed | NA | (N28XPA887P)PA887P | Mid Late | d0, d15, d30, d45, d60 and d75 |
| PHW79 | Oh7/Midland | PI601576 | PHT90 x 595 | Late | d0, d15, d30, d45, d60, d75 and d90 |

**Supplementary Table 1. Genotypes, origins, accession number, pedigree and visual senescence phenotype of the six genotypes used for the QTL transcription expression analysis.**

^X^ Origin described in Mikel & Dudley, 2006.

^y^ Visual phenotypes denote

^z^ Denote the RNA seq sampling time for each genotype as d0= flowering time, d15= 15 days after flowering, d30= 30 days after flowering, d45= 45 days after flowering, d60 = 60 days after flowering, d75= 75 days after flowering, d90= 90 days after flowering.

**Supplementary Table 2. QTLs mapped for visual senescence and SNP information with the genotype of the parents, allele effects, additive values, significance of the association, and proportion of the phenotypic variance explained by each SNP of the MAGIC population evaluated in Pontevedra, Spain.**

| **Trait** | **Time ^a^** | **SNP ^b^** | **A509** | **EP125** | **EP17** | **EP43** | **EP53** | **EP86** | **F473** | **PB130** | **Allele^c^** | **Add ^d^** | **p-value ^e^** | **R^2 f^** |
| --- | --- | --- | --- | --- | --- | --- | --- | --- | --- | --- | --- | --- | --- | --- |
| Visual | Silking | S2_216600055 | N | T | N | T | T | N | C | N | T/C | 0.074 | 1.82E-05 | 0.051 |
| Visual | Silking | S2_216600070 | N | C | N | C | C | N | T | N | C/T | 0.074 | 1.82E-05 | 0.051 |
| Visual | Silking | S3_156634936 | A | A | A | A | A | G | N | A | G/A | 0.057 | 2.37E-05 | 0.046 |
| Visual | Silking | S4_230352112 | N | A | N | C | C | A | C | N | C/A | 0.062 | 1.28E-05 | 0.077 |
| Visual | Silking | S4_231255536 | G | G | C | N | N | N | C | C | C/G | 0.052 | 1.74E-05 | 0.053 |
| Visual | Silking | S4_231255539 | A | A | G | N | N | N | G | G | G/A | 0.052 | 1.65E-05 | 0.053 |
| Visual | Silking | S4_231255542 | G | G | A | N | N | N | A | A | A/G | 0.052 | 1.65E-05 | 0.053 |
| Visual | Silking | S8_71975885 | G | G | G | N | N | N | G | G | G/A | 0.069 | 3.67E-06 | 0.076 |
| Visual | 2MAS | S3_8974495 | C | C | N | C | N | T | N | C | T/C | 0.16 | 9.06E-06 | 0.055 |
| Visual | 2MAS | S3_9038739 | G | G | N | N | G | A | N | G | A/G | 0.20 | 5.67E-06 | 0.088 |
| Visual | 2MAS | S6_32458889 | G | G | G | G | C | N | G | G | C/G | 0.24 | 9.09E-06 | 0.066 |
| Visual | 2MAS | S6_32895791 | C | N | C | N | T | N | C | C | T/C | 0.25 | 1.69E-05 | 0.057 |
| Visual | 2MAS | S6_32895795 | G | N | G | N | C | N | G | G | C/G | 0.25 | 1.69E-05 | 0.057 |
| Visual | 2MAS | S6_32895804 | A | N | A | N | T | N | A | A | T/A | 0.25 | 1.69E-05 | 0.057 |
| Visual | 2MAS | S6_32895805 | A | N | A | N | G | N | A | A | G/A | 0.25 | 1.69E-05 | 0.057 |
| Visual | 2MAS | S6_32895806 | T | N | T | N | C | N | T | T | C/T | 0.25 | 1.69E-05 | 0.057 |
| Visual | 2MAS | S6_32895807 | A | N | A | N | C | N | A | A | C/A | 0.25 | 1.69E-05 | 0.057 |
| Visual | 2MAS | S6_32895812 | A | N | A | N | C | N | A | A | C/A | 0.25 | 1.69E-05 | 0.057 |
| Visual | 2MAS | S6_32895814 | A | N | A | N | G | N | A | A | G/A | 0.25 | 1.75E-05 | 0.056 |
| Visual | 2MAS | S6_32895817 | G | N | G | N | T | N | G | G | T/G | 0.25 | 1.69E-05 | 0.057 |
| Visual | 2MAS | S6_32895818 | A | N | A | N | G | N | A | A | G/A | 0.25 | 1.69E-05 | 0.057 |
| Visual | 2MAS | S6_32895819 | A | N | A | N | C | N | A | A | C/A | 0.25 | 1.69E-05 | 0.057 |
| Visual | 2MAS | S6_32967880 | C | C | C | N | A | N | C | C | A/C | 0.24 | 1.53E-05 | 0.054 |
| Visual | 2MAS | S6_32967890 | C | C | C | N | T | N | C | C | T/C | 0.24 | 1.40E-05 | 0.057 |
| Visual | 2MAS | S6_32969296 | G | G | G | G | A | N | G | G | A/G | 0.22 | 2.16E-05 | 0.048 |
| Visual | 2MAS | S6_33178982 | G | N | G | N | A | G | G | G | A/G | 0.26 | 2.17E-05 | 0.059 |
| Visual | 2MAS | S6_33192007 | C | C | C | N | T | N | C | C | T/C | 0.23 | 2.32E-05 | 0.070 |
| Visual | 2MAS | S6_33434028 | A | A | A | A | G | N | A | A | G/A | 0.22 | 1.08E-05 | 0.046 |
| Visual | 2MAS | S6_33623678 | A | A | A | A | T | N | A | A | T/A | 0.25 | 1.26E-05 | 0.062 |
| Visual | 2MAS | S6_34123871 | C | C | C | C | T | C | N | C | T/C | 0.22 | 2.11E-05 | 0.048 |
| Visual | 2MAS | S6_34221026 | A | A | A | A | G | N | A | A | G/A | 0.25 | 3.60E-06 | 0.052 |
| Visual | 2MAS | S6_56810498 | A | A | N | A | G | A | G | N | G/A | 0.17 | 2.35E-05 | 0.051 |
| Visual | 2MAS | S7_127890381 | N | A | N | A | A | N | N | N | G/A | 0.31 | 5.54E-06 | 0.081 |
| Visual | 2MAS | S9_153511263 | N | G | G | G | N | N | A | G | A/G | 0.31 | 9.49E-06 | 0.064 |
| Visual | 2MAS | S10_2034863 | T | T | N | C | N | T | T | T | C/T | 0.21 | 1.46E-05 | 0.062 |

^a^ Evaluation time point at silking stage and two months after silking, 2MAS.

^b^ Chromosome number followed by the physical position of the SNP in base pairs.

^c^ First allele increased the trait, and the allele after / decreased the trait.

^d^ Additive allele effect substitution.

^e^ Significance threshold based on the deviation of F observed from expected.

^f^ R^2^ is the proportion of the phenotypic variance explained.

**Supplementary Table 3. QTLs mapped for chlorophyll and SNP information with the genotype of the parents, allele effects, additive values, significance of the association, and proportion of the phenotypic variance explained by each SNP of the MAGIC population evaluated in Pontevedra, Spain.**

| **Trait** | **Time ^a^** | **SNP ^b^** | **A509** | **EP125** | **EP17** | **EP43** | **EP53** | **EP86** | **F473** | **PB130** | **Allele^c^** | **Add ^d^** | **p-value ^e^** | **R^2 f^** |
| --- | --- | --- | --- | --- | --- | --- | --- | --- | --- | --- | --- | --- | --- | --- |
| Chlorophyl | Silking | S1_39067705 | A | C | A | C | N | N | N | N | A/C | 2.53 | 1.50E-05 | 0.083 |
| Chlorophyl | Silking | S1_49649566 | C | C | N | N | C | C | C | C | C/T | 4.24 | 1.55E-05 | 0.051 |
| Chlorophyl | Silking | S1_49649568 | T | T | N | N | T | T | T | T | T/G | 4.46 | 3.08E-06 | 0.063 |
| Chlorophyl | Silking | S1_49658620 | C | C | N | N | C | C | C | C | C/T | 3.78 | 3.16E-06 | 0.053 |
| Chlorophyl | Silking | S1_278163516 | N | T | C | T | C | N | C | C | C/T | 2.37 | 1.45E-05 | 0.061 |
| Chlorophyl | Silking | S1_278356935 | G | G | A | G | N | N | A | A | A/G | 2.29 | 5.23E-06 | 0.059 |
| Chlorophyl | Silking | S1_278992401 | N | A | A | A | A | A | G | G | G/A | 2.21 | 7.49E-06 | 0.047 |
| Chlorophyl | Silking | S3_181767075 | G | G | N | G | G | N | G | G | T/G | 4.19 | 2.24E-05 | 0.045 |
| Chlorophyl | Silking | S4_170805517 | N | G | A | G | N | N | N | N | A/G | 2.49 | 1.10E-05 | 0.071 |
| Chlorophyl | Silking | S4_170805550 | N | A | G | A | N | N | N | N | G/A | 2.49 | 1.46E-05 | 0.068 |
| Chlorophyl | Silking | S5_48503994 | A | N | N | G | G | G | G | G | G/A | 3.12 | 1.89E-05 | 0.054 |
| Chlorophyl | Silking | S5_48504499 | G | N | N | C | C | C | C | N | C/G | 3.33 | 8.92E-06 | 0.057 |
| Chlorophyl | Silking | S5_48504501 | T | N | N | C | C | C | C | N | C/T | 3.33 | 8.92E-06 | 0.057 |
| Chlorophyl | Silking | S5_48504503 | C | N | N | G | G | G | G | N | G/C | 3.33 | 8.92E-06 | 0.057 |
| Chlorophyl | Silking | S5_48504506 | G | N | N | T | T | T | T | N | T/G | 3.33 | 8.92E-06 | 0.057 |
| Chlorophyl | Silking | S5_174936437 | N | A | G | N | G | N | N | G | G/A | 2.20 | 9.41E-06 | 0.059 |
| Chlorophyl | Silking | S5_174936444 | N | A | T | N | T | N | N | T | T/A | 2.17 | 1.15E-05 | 0.058 |
| Chlorophyl | Silking | S5_175598960 | C | C | C | A | C | C | C | C | A/C | 2.77 | 2.01E-05 | 0.040 |
| Chlorophyl | Silking | S7_23234742 | G | N | C | N | C | N | G | C | G/C | 2.43 | 1.13E-05 | 0.053 |
| Chlorophyl | Silking | S8_155643923 | A | A | A | G | N | A | N | A | G/A | 2.42 | 2.21E-05 | 0.051 |
| Chlorophyl | Silking | S10_22131980 | G | G | N | G | G | G | G | G | C/G | 3.27 | 1.48E-05 | 0.047 |
| Chlorophyl | Silking | S10_80046122 | N | G | G | G | G | G | N | G | C/G | 4.55 | 4.47E-06 | 0.057 |
| Chlorophyl | Silking | S10_80046137 | N | G | G | G | G | G | N | G | T/G | 4.52 | 5.22E-06 | 0.056 |
| Chlorophyl | Silking | S10_80046138 | N | C | C | C | C | C | N | C | G/C | 4.43 | 6.30E-06 | 0.055 |
| Chlorophyl | Silking | S10_80046140 | N | C | C | C | C | C | N | C | A/C | 4.52 | 5.22E-06 | 0.056 |
| Chlorophyl | Silking | S10_80590477 | A | N | N | N | A | A | A | A | G/A | 5.00 | 8.36E-06 | 0.056 |
| Chlorophyl | Silking | S10_80616280 | C | C | C | C | C | C | C | C | T/C | 4.36 | 1.97E-05 | 0.044 |
| Chlorophyl | Silking | S10_81181498 | C | C | C | C | C | C | C | C | T/C | 3.97 | 2.27E-05 | 0.040 |
| Chlorophyl | Silking | S10_82041409 | A | A | A | A | A | A | A | A | G/A | 3.94 | 1.56E-05 | 0.042 |
| Chlorophyl | Silking | S10_85588442 | N | C | N | C | N | N | C | C | A/C | 4.66 | 9.08E-06 | 0.059 |
| Chlorophyl | Silking | S10_93396330 | T | T | C | N | N | T | T | T | C/T | 5.34 | 1.52E-06 | 0.065 |
| Chlorophyl | Silking | S10_99942058 | T | T | T | T | T | T | T | T | C/T | 3.99 | 1.11E-05 | 0.044 |
| Chlorophyl | 2MAS | S1_279901234 | C | T | T | C | T | T | C | C | C/T | 2.26 | 8.26E-07 | 0.084 |
| Chlorophyl | 2MAS | S1_279901243 | C | G | G | C | G | G | C | C | C/G | 2.26 | 8.26E-07 | 0.084 |
| Chlorophyl | 2MAS | S1_282899913 | G | G | N | G | G | G | G | N | A/G | 2.39 | 1.66E-05 | 0.047 |
| Chlorophyl | 2MAS | S3_196153899 | C | C | N | T | C | C | C | N | T/C | 3.46 | 8.24E-06 | 0.062 |
| Chlorophyl | 2MAS | S3_201194856 | G | G | A | G | G | A | G | G | A/G | 2.36 | 6.65E-06 | 0.045 |
| Chlorophyl | 2MAS | S3_201538092 | A | A | G | G | A | G | A | A | G/A | 1.96 | 3.82E-06 | 0.053 |
| Chlorophyl | 2MAS | S3_201538103 | A | A | G | G | A | G | A | A | G/A | 1.91 | 7.17E-06 | 0.051 |
| Chlorophyl | 2MAS | S3_201538104 | C | C | T | T | C | T | C | C | T/C | 1.96 | 3.82E-06 | 0.053 |
| Chlorophyl | 2MAS | S3_201538113 | A | A | G | G | A | G | A | A | G/A | 1.96 | 3.82E-06 | 0.053 |
| Chlorophyl | 2MAS | S3_208904821 | C | N | N | C | N | G | N | C | G/C | 3.68 | 4.03E-06 | 0.073 |
| Chlorophyl | 2MAS | S3_208904825 | A | N | N | A | N | G | N | A | G/A | 3.67 | 4.35E-06 | 0.073 |
| Chlorophyl | 2MAS | S4_106631292 | G | G | G | G | G | N | N | G | A/G | 3.85 | 1.78E-05 | 0.078 |
| Chlorophyl | 2MAS | S4_108425620 | T | T | T | N | T | T | N | T | G/T | 3.71 | 1.62E-05 | 0.056 |
| Chlorophyl | 2MAS | S4_150637753 | T | N | N | N | T | N | T | T | C/T | 3.85 | 4.85E-06 | 0.065 |
| Chlorophyl | 2MAS | S4_150637846 | G | G | N | N | G | N | N | G | A/G | 3.75 | 8.15E-06 | 0.061 |
| Chlorophyl | 2MAS | S5_58599162 | N | G | G | G | G | G | G | G | A/G | 2.17 | 1.30E-05 | 0.047 |
| Chlorophyl | 2MAS | S5_174114678 | A | A | A | N | A | N | A | A | T/A | 3.97 | 1.85E-05 | 0.061 |
| Chlorophyl | 2MAS | S5_174114682 | G | G | G | N | G | N | G | G | C/G | 3.97 | 1.85E-05 | 0.061 |
| Chlorophyl | 2MAS | S6_93190538 | G | N | N | N | A | N | N | G | A/G | 2.51 | 1.63E-06 | 0.087 |

^a^ Evaluation time point at silking stage and two months after silking, 2MAS.

^b^ Chromosome number followed by the physical position of the SNP in base pairs.

^c^ First allele increased the trait, and the allele after / decreased the trait.

^d^ Additive allele effect substitution.

^e^ Significance threshold based on the deviation of F observed from expected.

^f^ R^2^ is the proportion of the phenotypic variance explained.

**Supplementary Table 4. QTLs mapped for PSII quenching and SNP information with the genotype of the parents, allele effects, additive values, significance of the association, and proportion of the phenotypic variance explained by each SNP of the MAGIC population evaluated in Pontevedra, Spain.**

| **Trait** | **Time ^a^** | **SNP ^b^** | **A509** | **EP125** | **EP17** | **EP43** | **EP53** | **EP86** | **F473** | **PB130** | **Allele^c^** | **Add ^d^** | **p-value ^e^** | **R^2 f^** |
| --- | --- | --- | --- | --- | --- | --- | --- | --- | --- | --- | --- | --- | --- | --- |
| PSII | Silking | S1_45426763 | C | C | C | A | C | N | C | C | C/A | 6.56 | 3.83E-06 | 0.050 |
| PSII | Silking | S1_45771866 | T | T | T | C | C | T | T | T | T/C | 6.18 | 5.87E-06 | 0.052 |
| PSII | Silking | S1_45771867 | A | A | A | C | C | A | A | A | A/C | 6.18 | 5.87E-06 | 0.052 |
| PSII | Silking | S1_45771877 | A | A | A | G | G | A | A | A | A/G | 6.21 | 5.77E-06 | 0.052 |
| PSII | Silking | S1_45938671 | A | A | A | T | A | A | N | A | A/T | 6.73 | 4.54E-06 | 0.051 |
| PSII | Silking | S1_46841551 | A | G | G | A | G | G | G | A | G/A | 4.97 | 2.20E-05 | 0.042 |
| PSII | Silking | S1_47177423 | A | A | N | T | T | N | N | A | A/T | 5.54 | 1.92E-05 | 0.046 |
| PSII | Silking | S1_47331494 | A | A | N | T | T | N | A | N | A/T | 5.58 | 2.32E-05 | 0.050 |
| PSII | Silking | S1_47455786 | C | C | N | T | T | C | C | T | C/T | 5.55 | 3.61E-06 | 0.050 |
| PSII | Silking | S1_47599783 | N | A | T | A | A | T | T | A | T/A | 6.03 | 6.29E-06 | 0.061 |
| PSII | Silking | S1_47599785 | N | C | T | C | C | T | T | C | T/C | 6.03 | 6.29E-06 | 0.061 |
| PSII | Silking | S1_47600003 | G | A | G | A | A | G | G | A | G/A | 5.27 | 7.23E-06 | 0.046 |
| PSII | Silking | S1_47705163 | T | T | C | T | T | C | C | T | C/T | 5.25 | 8.12E-06 | 0.046 |
| PSII | Silking | S1_47775156 | G | G | N | T | T | N | G | T | G/T | 5.62 | 9.35E-06 | 0.054 |
| PSII | Silking | S1_50288007 | G | G | G | A | G | N | N | N | G/A | 8.70 | 7.63E-06 | 0.083 |
| PSII | Silking | S1_50421412 | N | G | G | A | N | N | N | G | G/A | 8.28 | 1.28E-05 | 0.057 |
| PSII | Silking | S4_200030889 | G | G | C | G | N | G | G | N | G/C | 7.15 | 1.85E-05 | 0.072 |
| PSII | Silking | S6_128564797 | T | N | T | G | T | T | N | T | T/G | 10.16 | 2.11E-05 | 0.079 |
| PSII | Silking | S10_105878718 | A | A | N | G | A | N | N | N | A/G | 7.61 | 3.57E-06 | 0.072 |
| PSII | 2MAS | S3_207022252 | N | G | N | G | N | N | G | G | A/G | 79.98 | 1.56E-05 | 0.054 |
| PSII | 2MAS | S6_162496073 | G | G | T | G | N | G | N | G | T/G | 103.79 | 2.34E-05 | 0.055 |

^a^ Evaluation time point at silking stage and two months after silking, 2MAS.

^b^ Chromosome number followed by the physical position of the SNP in base pairs.

^c^ First allele increased the trait, and the allele after / decreased the trait.

^d^ Additive allele effect substitution.

^e^ Significance threshold based on the deviation of F observed from expected.

^f^ R^2^ is the proportion of the phenotypic variance explained.

**Supplementary Table 5. QTLs mapped for chlorophyll fluorescence (F0) and SNP information with the genotype of the parents, allele effects, additive values, significance of the association, and proportion of the phenotypic variance explained by each SNP of the MAGIC population evaluated in Pontevedra, Spain.**

| **Trait** | **Time ^a^** | **SNP ^b^** | **A509** | **EP125** | **EP17** | **EP43** | **EP53** | **EP86** | **F473** | **PB130** | **Allele^c^** | **Add ^d^** | **p-value ^e^** | **R^2 f^** |
| --- | --- | --- | --- | --- | --- | --- | --- | --- | --- | --- | --- | --- | --- | --- |
| F0 | Silking | S1_10971254 | G | G | G | A | G | N | G | G | A/G | 2.36 | 1.64E-05 | 0.060 |
| F0 | Silking | S1_18190570 | G | G | G | A | N | G | N | N | A/G | 2.08 | 8.84E-06 | 0.061 |
| F0 | Silking | S1_21703366 | C | C | N | G | C | C | C | C | G/C | 1.81 | 2.21E-05 | 0.047 |
| F0 | Silking | S1_21714481 | G | G | N | N | G | N | N | N | T/G | 1.94 | 4.86E-06 | 0.062 |
| F0 | Silking | S1_21714543 | T | T | N | A | T | N | T | A | A/T | 1.80 | 6.13E-06 | 0.054 |
| F0 | Silking | S1_21758984 | C | C | C | A | C | C | N | C | A/C | 2.56 | 1.35E-08 | 0.085 |
| F0 | Silking | S1_24673475 | A | C | A | C | A | A | A | A | C/A | 1.84 | 1.90E-07 | 0.073 |
| F0 | Silking | S1_25104823 | G | N | G | A | G | G | G | G | A/G | 1.88 | 1.35E-07 | 0.066 |
| F0 | Silking | S1_25187529 | T | T | T | G | T | T | T | T | G/T | 1.79 | 2.06E-06 | 0.056 |
| F0 | Silking | S1_25287700 | T | T | T | C | T | T | T | T | C/T | 2.25 | 1.19E-06 | 0.067 |
| F0 | Silking | S1_25745017 | T | T | T | C | T | N | T | N | C/T | 1.44 | 2.10E-05 | 0.063 |
| F0 | Silking | S1_25882412 | C | C | C | N | N | N | C | N | T/C | 1.64 | 1.19E-05 | 0.064 |
| F0 | Silking | S1_25882429 | G | G | G | N | N | N | G | N | A/G | 1.64 | 1.19E-05 | 0.064 |
| F0 | Silking | S1_25882437 | G | G | G | N | N | N | G | N | T/G | 1.64 | 1.19E-05 | 0.064 |
| F0 | Silking | S1_26037555 | G | G | G | T | G | G | G | G | T/G | 1.81 | 1.34E-07 | 0.067 |
| F0 | Silking | S1_26037640 | A | N | A | G | A | N | A | A | G/A | 1.77 | 7.86E-07 | 0.068 |
| F0 | Silking | S1_26037654 | G | N | G | T | G | N | G | G | T/G | 1.77 | 8.44E-07 | 0.068 |
| F0 | Silking | S1_26037694 | G | G | G | T | G | N | G | G | T/G | 1.63 | 6.11E-06 | 0.059 |
| F0 | Silking | S1_26037701 | G | G | G | A | G | N | G | G | A/G | 1.63 | 6.11E-06 | 0.059 |
| F0 | Silking | S1_26037715 | T | T | T | G | T | N | T | T | G/T | 1.63 | 6.11E-06 | 0.059 |
| F0 | Silking | S1_26038293 | C | C | N | A | C | N | C | C | A/C | 1.73 | 1.76E-06 | 0.068 |
| F0 | Silking | S1_26038329 | A | A | N | T | A | N | A | A | T/A | 1.73 | 1.76E-06 | 0.068 |
| F0 | Silking | S1_26039897 | A | A | N | G | A | N | A | A | G/A | 1.68 | 9.21E-06 | 0.065 |
| F0 | Silking | S1_26690897 | G | N | N | A | G | G | G | G | A/G | 1.57 | 3.75E-06 | 0.059 |
| F0 | Silking | S1_26692540 | C | N | N | N | N | N | N | N | A/C | 1.98 | 1.96E-06 | 0.108 |
| F0 | Silking | S1_26693647 | G | N | N | N | N | N | N | N | A/G | 1.88 | 8.69E-06 | 0.095 |
| F0 | Silking | S1_26694070 | C | N | N | A | N | N | N | N | A/C | 1.77 | 1.42E-05 | 0.073 |
| F0 | Silking | S1_26694075 | A | N | N | C | N | N | N | N | C/A | 1.77 | 1.42E-05 | 0.073 |
| F0 | Silking | S1_26694078 | A | N | N | T | N | N | N | N | T/A | 1.77 | 1.42E-05 | 0.073 |
| F0 | Silking | S1_26694079 | A | N | N | C | N | N | N | N | C/A | 1.77 | 1.42E-05 | 0.073 |
| F0 | Silking | S1_26694115 | G | N | N | T | N | N | N | N | T/G | 1.77 | 1.42E-05 | 0.073 |
| F0 | Silking | S1_35133728 | G | C | G | G | G | G | G | G | C/G | 2.36 | 1.25E-05 | 0.048 |
| F0 | Silking | S1_254098677 | C | C | C | N | C | C | C | C | T/C | 2.34 | 1.10E-05 | 0.055 |
| F0 | Silking | S1_254098687 | C | C | C | N | C | C | C | C | T/C | 2.34 | 1.10E-05 | 0.055 |
| F0 | Silking | S1_291102452 | A | G | A | N | G | A | G | N | A/G | 1.20 | 1.28E-05 | 0.067 |
| F0 | Silking | S2_160962926 | C | C | C | C | T | C | C | C | T/C | 2.43 | 3.56E-06 | 0.060 |
| F0 | Silking | S2_187816927 | C | C | C | C | N | C | N | C | C/T | -1.24 | 1.63E-05 | 0.045 |
| F0 | Silking | S3_49149279 | T | T | T | C | C | T | T | N | C/T | 1.21 | 3.83E-06 | 0.050 |
| F0 | Silking | S3_144742460 | T | T | N | N | N | N | C | C | T/C | 1.10 | 1.85E-05 | 0.060 |
| F0 | Silking | S3_227868893 | T | T | T | A | A | T | T | N | A/T | 1.34 | 1.65E-05 | 0.067 |
| F0 | Silking | S5_100157583 | T | N | N | G | G | N | N | G | T/G | 1.20 | 2.44E-06 | 0.060 |
| F0 | Silking | S6_146568385 | G | G | A | N | G | N | G | G | A/G | 1.36 | 1.02E-05 | 0.055 |
| F0 | Silking | S7_4958091 | G | C | G | C | G | G | G | G | C/G | 1.27 | 2.41E-05 | 0.043 |
| F0 | 2MAS | S3_200700872 | A | A | N | A | A | C | A | A | C/A | 9.68 | 1.14E-05 | 0.047 |
| F0 | 2MAS | S3_200877582 | G | G | N | G | G | A | N | G | A/G | 10.42 | 1.09E-05 | 0.059 |
| F0 | 2MAS | S3_201049765 | A | A | N | A | N | G | A | A | G/A | 10.35 | 1.12E-05 | 0.053 |
| F0 | 2MAS | S3_201049777 | A | A | N | A | N | G | A | A | G/A | 10.35 | 1.12E-05 | 0.053 |
| F0 | 2MAS | S3_201049786 | A | A | N | A | N | G | A | A | G/A | 10.35 | 1.12E-05 | 0.053 |
| F0 | 2MAS | S3_201538092 | A | A | G | G | A | G | A | A | G/A | 6.82 | 1.63E-05 | 0.046 |
| F0 | 2MAS | S3_201538103 | A | A | G | G | A | G | A | A | G/A | 7.34 | 3.93E-06 | 0.056 |
| F0 | 2MAS | S3_201538104 | C | C | T | T | C | T | C | C | T/C | 6.82 | 1.63E-05 | 0.046 |
| F0 | 2MAS | S3_201538113 | A | A | G | G | A | G | A | A | G/A | 6.82 | 1.63E-05 | 0.046 |
| F0 | 2MAS | S3_201538222 | G | G | N | N | G | G | G | G | T/G | 8.87 | 2.02E-06 | 0.063 |
| F0 | 2MAS | S3_206319665 | T | T | N | T | N | C | T | N | C/T | 10.28 | 6.92E-06 | 0.074 |
| F0 | 2MAS | S3_207022252 | N | G | N | G | N | N | G | G | A/G | 10.23 | 2.93E-06 | 0.065 |
| F0 | 2MAS | S3_208613140 | C | C | C | C | T | C | N | N | T/C | 11.27 | 5.47E-06 | 0.060 |
| F0 | 2MAS | S3_208904821 | C | N | N | C | N | G | N | C | G/C | 13.57 | 4.74E-06 | 0.083 |
| F0 | 2MAS | S3_208904825 | A | N | N | A | N | G | N | A | G/A | 13.54 | 5.21E-06 | 0.084 |
| F0 | 2MAS | S3_208904958 | G | G | N | G | G | A | G | G | A/G | 11.78 | 6.46E-06 | 0.055 |
| F0 | 2MAS | S3_208904976 | C | C | N | C | C | T | C | C | T/C | 11.78 | 6.46E-06 | 0.055 |
| F0 | 2MAS | S3_223086559 | A | A | N | A | N | G | N | A | G/A | 14.61 | 1.45E-05 | 0.070 |
| F0 | 2MAS | S5_212693257 | T | T | T | T | T | A | N | N | T/A | 12.55 | 1.10E-05 | 0.067 |
| F0 | 2MAS | S8_148315035 | G | G | G | G | G | N | A | G | A/G | 10.12 | 2.20E-05 | 0.049 |

^a^ Evaluation time point at silking stage and two months after silking, 2MAS.

^b^ Chromosome number followed by the physical position of the SNP in base pairs.

^c^ First allele increased the trait, and the allele after / decreased the trait.

^d^ Additive allele effect substitution.

^e^ Significance threshold based on the deviation of F observed from expected.

^f^ R^2^ is the proportion of the phenotypic variance explained.
