# Supplementary figures and images for: Precision Mapping of a Maize MAGIC Population Identified a Candidate Gene for the Senescence-Associated Physiological Traits
Source: Front Genet. 2021 Oct 4;12:716821. doi: 10.3389/fgene.2021.716821 (PMC8521056; doi:10.3389/fgene.2021.716821)

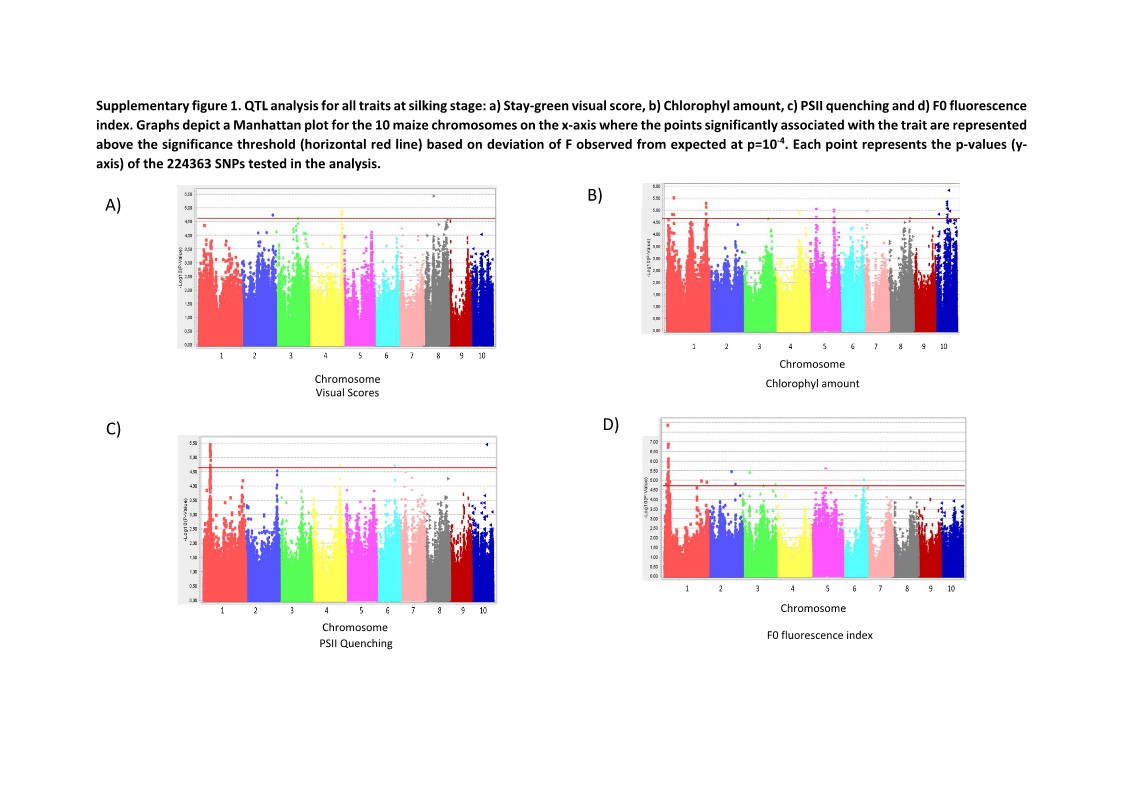

Supplement: Supplementary file 2 [file Image_1.jpeg]

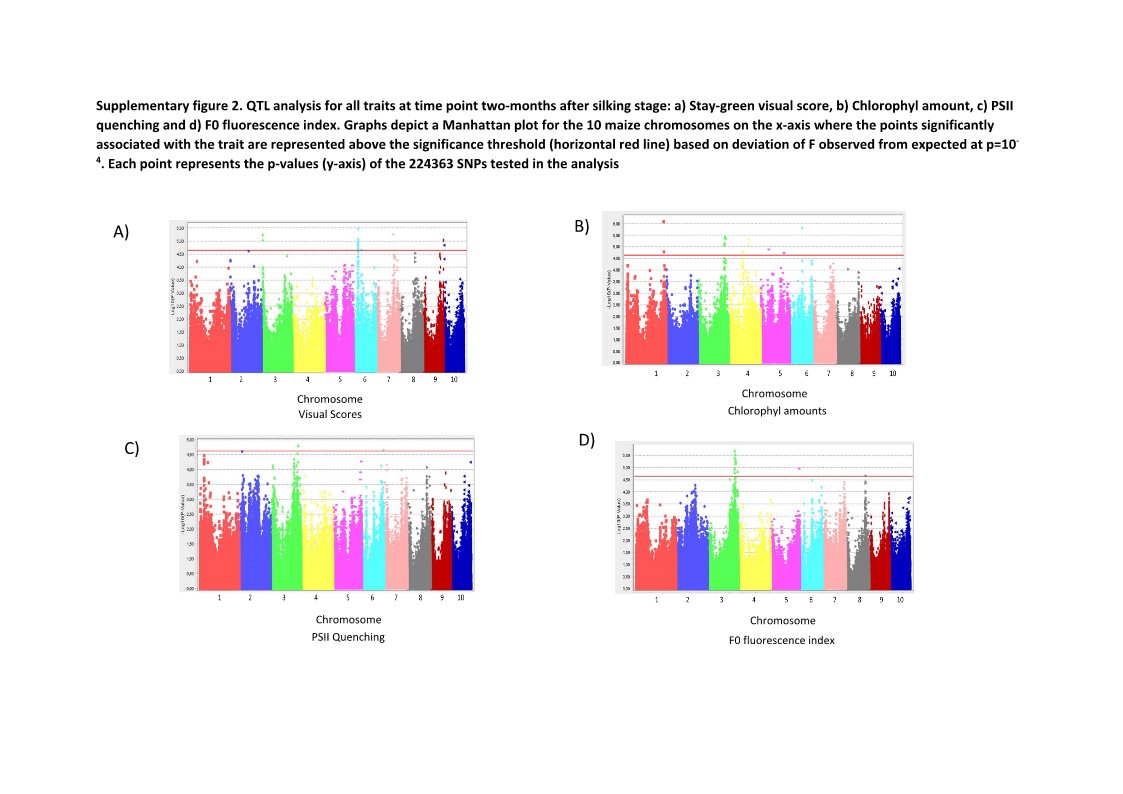

Supplement: Supplementary file 3 [file Image_2.jpeg]
